# Research in disaster settings: a systematic qualitative review of ethical guidelines

**DOI:** 10.1186/s12910-016-0148-7

**Published:** 2016-10-21

**Authors:** Signe Mezinska, Péter Kakuk, Goran Mijaljica, Marcin Waligóra, Dónal P. O’Mathúna

**Affiliations:** 1 Faculty of Medicine, University of Latvia, Riga, Latvia; 2 Department of Behavioural Sciences, Faculty of Public Health, University of Debrecen, Debrecen, Hungary; 3 Department of Medical Humanities, University of Split School of Medicine, Split, Croatia; 4 Department of Philosophy and Bioethics, REMEDY, Research Ethics in Medicine Study Group, Jagiellonian University Medical College, Kraków, Poland; 5 School of Nursing and Human Sciences, Dublin City University, Dublin, Ireland

**Keywords:** Disaster, Disaster research, Ethics guidelines, Research ethics, Research ethics committee, Vulnerability

## Abstract

**Background:**

Conducting research during or in the aftermath of disasters poses many specific practical and ethical challenges. This is particularly the case with research involving human subjects. The extraordinary circumstances of research conducted in disaster settings require appropriate regulations to ensure the protection of human participants. The goal of this study is to systematically and qualitatively review the existing ethical guidelines for disaster research by using the constant comparative method (CCM).

**Methods:**

We performed a systematic qualitative review of disaster research ethics guidelines to collect and compare existing regulations. Guidelines were identified by a three-tiered search strategy: 1) searching databases (PubMed and Google Scholar), 2) an Internet search (Google), and 3) a search of the references in the included documents from the first two searches. We used the constant comparative method (CCM) for analysis of included guidelines.

**Results:**

Fourteen full text guidelines were included for analysis. The included guidelines covered the period 2000-2014. Qualitative analysis of the included guidelines revealed two core themes: vulnerability and research ethics committee review. Within each of the two core themes, various categories and subcategories were identified.

**Conclusions:**

Some concepts and terms identified in analyzed guidelines are used in an inconsistent manner and applied in different contexts. Conceptual clarity is needed in this area as well as empirical evidence to support the statements and requirements included in analyzed guidelines.

**Electronic supplementary material:**

The online version of this article (doi:10.1186/s12910-016-0148-7) contains supplementary material, which is available to authorized users.

## Background

Disasters are defined as phenomena caused by environmental events or armed conflicts that lead to fatalities, injuries, stress, physical damage and economic breakdown of great significance [1, 2]. They occur on a scale that overwhelms local resources, usually requiring external assistance. Improving the effectiveness and efficiency of interventions, and the fairness of their distribution, is crucial in the field of disaster response. For that reason, increasing and improving the scientific evidence for disaster relief is essential. Research is also vital to accurately describe phenomena in disasters, also called humanitarian emergencies or crises [3–5]. Conducting research during or in the aftermath of disasters poses many specific practical and ethical challenges. This is particularly the case with research involving human subjects where data collection must be balanced with the appropriate protection of research subjects. Researchers play a central part in this analysis, as does the system of research ethics review. Such a system, involving research ethics committees (RECs) or institutional review boards (IRBs), is crucial to ensure compliance with existing international and national standards and more general principles of research ethics. The extraordinary circumstances of research conducted in disaster settings require appropriate regulations to ensure the protection of human participants. We decided to perform a systematic qualitative review of existing disaster research ethics guidelines to collect and compare existing regulations. The goal of this study is to systematically and qualitatively review the existing ethical guidelines for disaster research using the constant comparative method (CCM).

## Methods

### Search strategy

We identified guidelines for research ethics in disaster situations by a three-tiered search strategy: 1) searching two databases (PubMed and Google Scholar), 2) an Internet search (Google), and 3) a search of the references in the documents included from the first two searches. We used the following search terms: (guidelines AND “research ethics” AND (disaster OR emergency OR crisis)). Assessment of eligibility was limited to the first 200 hits retrieved in Google and to the first 250 hits in Google Scholar ordered by relevance in accordance with the methods used in numerous similar systematic reviews. Limits were not placed on the PubMed search.

**Fig. 1:**
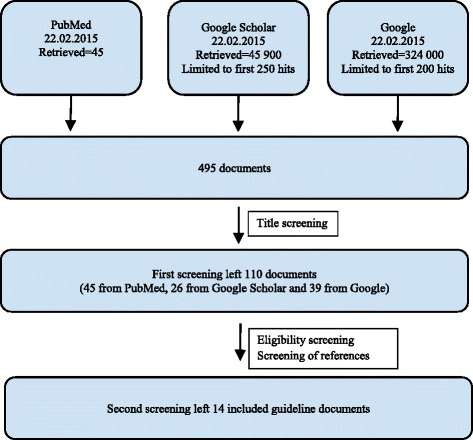
Screening process for identified articles

The screening process is summarized in Fig. 1. At the first screening stage, one researcher reviewed the document titles. Only documents written in English or translated into English by the guideline developers were included. Titles clearly not related to the topic, as well as scientific and popular articles, books, presentations, and opinion pieces which were clearly not guidelines were excluded. This gave 110 documents which were further screened. The second eligibility screening was performed independently by two researchers. Each researcher evaluated the documents against the inclusion criteria and screened the document’s reference list for additional disaster research ethics guidelines. Independent results were compared between the two researchers. When discrepancies existed, a third researcher was involved to resolve any eligibility disagreements.

### Inclusion/exclusion criteria

Documents were included if they fulfilled all the following criteria:

A. satisfied our definition of research ethics guidelines: systematically developed statements to assist with the responsible conduct of researchers and other stakeholders in the process of planning, conducting, and reporting research;
B. was issued by an international or national organization/institution/meeting or developed by a group of researchers or an individual researcher;
C. research ethics in disaster settings was addressed in the whole document or at least in its own part or section;
D. addressed at least one of the following types of research: clinical drug research; biomedical research involving physical interventions; public health research; research on health data or biological material; psychological or social sciences research.

### Qualitative analysis

We used the constant comparative method (CCM) for analysis of included guidelines [6, 7]. This method of qualitative analysis combines inductive category coding with a simultaneous comparison of all obtained units of meaning. A unit of meaning is defined as a part of the text (e.g., phrase, sentence, several sentences) that “must be understandable without additional information, except for knowledge of the researcher’s focus of inquiry” [7]. Open coding was applied as a first step in the coding process to identify units of meaning and to allow categories to emerge from the data. According to CCM, each new unit of meaning is “compared to all other units and subsequently grouped (categorized and coded) with similar units of meaning” [7]. In the process of open coding and comparison, initial categories were changed, merged and omitted when necessary. The second step involved axial coding to explore connections between categories and subcategories. Selective coding as a third step involved selecting the core themes. As a result of selective coding, we identified two core themes emerging from our analysis: research ethics review process and vulnerability.

## Results

The research team reached consensus on including 14 full text guidelines for analysis (see Additional file 1: Table S1 for the complete list). The included guidelines were published during the period 2000-2014, with a peak in the number of documents being issued between 2008 and 2010 (8 out of 14 guidelines were published during this period).

### Issuing organizations

The guidelines identified during the search showed a diversity in geography and types of issuing organizations. Seven out of 14 guidelines are applicable internationally, five are national level guidelines (two each from Canada and the US, and one from India), and two apply to a particular organization (Médecins Sans Frontières (MSF) or Doctors Without Borders) or institution (US Centers for Disease Control and Prevention). However, it should be taken into account that only guidelines written in English were included, which is a limitation of this study.

International organizations issuing guidelines included the World Health Organization (WHO) [8], MSF [9] and the International NGO Training and Research Centre [10]. Several national organizations issued guidelines, including the Indian Council of Medical Research [11] and the Canadian Medical Association [12]. Three guidelines arose from particular meetings or specific working groups including: a meeting entitled “Ethical Issues Pertaining to Research in the Aftermath of Disaster,” organized by the New York Academy of Medicine and the National Institute of Mental Health [13]; the Harvard Humanitarian Action Summit [14]; and the Working Group on Disaster Research and Ethics [15].

### Content of the guidelines

Qualitative analysis of the 14 included guidelines revealed two core themes: vulnerability and REC review process. Much of the material addressed in the guidelines could be grouped under one of these themes. At the same time, the themes overlap and a clear distinction between the two is not possible. Within each of the two core themes, various categories were identified, and within each of these, further subcategories were identified. These are summarized in Additional file 2: Table S2 and Additional file 3: Table S3, and described in detail in the sections below.

#### Vulnerability

We discovered four main categories applying to vulnerability of participants: vulnerability as a concept; risks and burdens; risk management; decisional capacity of research subjects. For each category, we identified a set of subcategories. The full list of categories and subcategories is presented in Additional file 2: Table S2 and referenced to the specific guidelines.

### Vulnerability as a concept

We identified three subcategories within this category: definitions of vulnerability (in four guidelines), reasons for vulnerability (in six guidelines), and gaps in the existing guidelines (in two guidelines).

Four out of 14 guidelines included a definition of vulnerability or analysed the concept of vulnerability. For example, Collogan et al. discussed different approaches to the definition of vulnerability and contrasted vulnerability as “a characteristic of the group” with “certain individual characteristics” [13]. The authors of this guideline criticized very broad definitions of vulnerability because these can be applied “to cover almost any person, group, or situation” and often serve to stereotype and disempower research participants [13].

Six guidelines mentioned and analysed the reasons for vulnerability in disaster settings. These arose from specific disaster situations and included, for example, political status and human rights abuses in refugee populations [16]; young and old age of research subjects, social vulnerability, physical injuries, and experience of violent and traumatic events leading to mental health problems in the aftermath of disaster [13]; psychological and physical consequences of a disaster, as well as poverty in pre-disaster settings and disempowerment in post-disaster settings [17], increased public risks and devastation [18] and substantial psychological stress in humanitarian settings [19].

Two guidelines issued in the early 2000s pointed to gaps in other general research ethics guidelines (such as the Declaration of Helsinki, Council for International Organizations of Medical Sciences (CIOMS) guidelines, etc.) and noted that these existing guidelines look at vulnerability very generally and do not address the special circumstances of disaster settings [17] or do not provide an applicable definition of vulnerability [13, 16].

### Risks and burdens

This category covered different types of risks and burdens that research subjects might face during disaster research. The category included six subcategories: physical harm (in seven guidelines), re-traumatization (in five guidelines), manipulation (in two guidelines), exploitation (in eight guidelines), unrealistic expectations (in eight guidelines) and stigmatization (in two guidelines).

Various guidelines mentioned different sources of possible direct physical harm to research subjects, depending of the type of research study and the authors’ experience. Goodhand mentioned a possibility that research interventions might “affect the incentives system and structures driving violent conflict or impact upon the coping strategies and safety of communities” [10]. Leaning referred to situations where “sampling techniques may confer unintended negative attention or focus on particularly vulnerable subpopulations […] and make conditions worse” [16]. Allden et al. mentioned several examples of physical risks, e.g., the fact that “the presence of western researchers in itself, could present a danger in targeting of local civilians” [14]. The authors gave an example where “researchers gave great attention to ethics and staff behaviour only to learn subsequently that the research group’s driver, who stayed with the research staff in the village where research was being conducted, engaged in transactional sex with girls <18 years of age” [14]. Tansey et al. paid attention to physical risks posed by research involving novel interventions and suggested that, “Early detection of toxicities or other harmful effects of a research intervention could help to reduce research-related harms by leading to adjustments in protocol or, if necessary, termination of the intervention” [12]. Collogan et al. and Curry et al. provided the general term “physical risks” and did not elaborate on particular types of risks or reasons for them [13, 19].

Guidelines were more detailed in their analysis of different types of non-physical risks. The most often mentioned risks were exploitation of research subjects and unrealistic expectations, as well as manipulation. While some guidelines just mentioned exploitation in general, others gave more detailed examples, e.g., Schopper et al. mentioned the collection, export, and analysis of tissues as a potential source of “exploitation of communities from which tissues have been taken” [20]. Allden et al. paid attention to children as a vulnerable group “who could be targeted for recruitment by groups that could exploit them as a result of research and program activities” [14]. Sumathipala et al. expressed deep concerns regarding possible exploitation and referred specifically to “exploitation through undue inducement and compensation, and through an understandable confusion regarding the researchers’ objectives” [15]. This type of exploitation leads into the next subcategory: unrealistic expectations. This was analysed broadly, with the general advice that researchers should be “particularly vigilant in ensuring that prospective study participants do not confuse research procedures with clinical care and evaluation and thus fall prey to the so-called therapeutic misconception” [13] and to “take great care to inform potential research participants […] that some interventions to which they are subjected might be undertaken primarily for the benefit of the research” [21].

The risk of stigmatization was mentioned less often, being named in two guidelines. Allden et al. emphasized that vulnerable groups might be stigmatized after participation in research, but individual research participants might be stigmatized by researchers who lack cultural competencies and knowledge of specific socio-cultural contexts [14]. Sumathipala et al. paid attention to researchers’ duty to prevent stigmatization [15].

Another risk, mentioned in five guidelines, was re-traumatization of research participants. Goodhand explained that by involving subjects in a research study and by asking sensitive questions “researchers may inadvertently re-open wounds by probing into areas respondents may not wish to talk about” [10]. Therefore, RECs should assess the risk of re-traumatization [20]. Specific ways of addressing re-traumatisation were mentioned in some guidelines, such as Sumathipala et al. describing the campaign “Prevent Re-traumatisation of the Traumatised” as an example of good practice [15]. Some guidelines noted the importance of recognising that disaster settings offer “limited opportunities for therapeutic interventions to handle adverse psychological reactions” [17]. At the same time, some authors argued that “an individual noted to be upset during participation in research might not necessarily regret participation” [13].

### Risk management

Taking into account the vulnerability of research participants leads to a need to manage risks. The risk management category included six subcategories: accountability and monitoring of research (in nine guidelines); avoiding over- or underestimation of risks (in three guidelines); the need for empirical evidence on risk (in three guidelines); providing psychological support to research subjects (in four guidelines); quality of informed consent (in six guidelines); and evaluation of power relationships between researchers and subjects (in two guidelines).

Accountability and monitoring of research was covered by eight guidelines. Authors of these guidelines, especially after 2008, emphasized that risks in disaster research can be diminished by monitoring and control [13] and mentioned corresponding values, e.g., accountability and transparency [8]. The most detailed description of monitoring was developed by Curry et al. [19]. However, most guidelines did not clearly describe what monitoring and control should include and what institutions should oversee the research studies. Direct oversight of the whole process of research implementation was mentioned in some guidelines, but seen as practically impossible [20]; however, close monitoring of particular parts of the process, e.g., informed consent procedures [12], was viewed as necessary. Some guidelines mentioned that new information arising during the research should be carefully monitored, e.g., protocol amendments, side effects, adverse effects and early stopping of a study. A different approach was to increase monitoring of specific types of research “where risk is high or uncertain” [12]. Various monitoring bodies were proposed, with some guidelines suggesting that monitoring could be done “by the central IRB or a separate data and safety monitoring board” [17].

Some guidelines noted that preliminary risk assessment should avoid both over- and underestimation of risks, e.g., overestimation by labelling all research participants as ‘vulnerable’ [21] or misusing the concept of ‘re-traumatization’ [13], and underestimation of risks by denying that research might add additional risks to those posed by the disaster [17]. The same guidelines dealing with estimation of risks mentioned the need for empirical evidence to evaluate risks posed by research [13, 17, 21].

One possible additional approach to risk management mentioned in four guidelines was ensuring psychological support to research subjects, including training of researchers and development of procedures to provide psychological support [21], as well as “explicit mechanisms available for timely referral of subjects in need of mental health consultation” [13]. These support mechanisms should be culturally and politically acceptable [14]. Other guidelines emphasized the need to identify available local services and to help research participants access these services when needed [17].

Another subcategory of risk management was the quality of informed consent, evaluated by some authors as one of the major ethical challenges in disaster research [20]. Examples of shortcomings in informed consent included “incomplete information given to the participants about objectives, risks, adverse effects, and planned house visits; information too detailed and complicated; formulation of the text biased to induce a positive answer; overestimation of the benefit for participants and community; and lack of procedures to ensure that the information provided is understood” [20]. To ensure higher quality informed consent, the guidelines included criteria usually mentioned by general research ethics guidelines, as well as criteria specific to disaster settings. These specific criteria emphasized the impact of vulnerability on research subjects and the safety of the setting where informed consent procedures take place [13]. Allden et al. suggested that researchers should “take consent at multiple times during the research process, including at the end of data collection” and “take consent from multiple agencies including community, parents, and partners as appropriate” [14].

An additional subcategory of risk management was mentioned in two guidelines: the evaluation of power relationships between researchers and subjects [10, 14]. Allden et al. stated that in disaster settings researchers hold more power than participants and therefore researchers should “be aware of power differentials between the researcher and respondent that may increase their likelihood of participation” [14].

### Decisional capacity of research subjects

We identified three subcategories for this category: factors diminishing decisional capacity (in four guidelines); underestimation of decisional capacity (in two guidelines); and the need for a specific procedure for informed consent (in five guidelines). Two guidelines mentioned the traumatic experiences of research participants [13, 21] and one guideline referred to “inherent tensions and pressures” in situations of public emergencies [18] as a possible reason for impaired decision-making capacity. To take account of this, a specific procedure for informed consent was proposed that would address diminished decisional capacity of research participants in disaster settings. This specific procedure should involve “a time lag between an initial contact and eventual interview” [21], “language specific to the unique situation of protection of victims of disasters as well as their communities” [17], and use of oral instead of written consent in cases when participants decline to sign anything [20]. At the same time, authors of several guidelines warned about possible underestimation of decisional capacity of participants by stating that it would “be inaccurate and potentially stigmatizing to assume that all persons who have experienced terror or other disasters are decisionally impaired and unable to make choices for themselves” [13].

#### Research ethics committee (REC) review process

The second core theme identified by our qualitative analysis was REC review procedures and processes. Research in disaster settings raises specific ethical concerns around review, with frequent calls for a different approach to review than typically conducted with other types of research. During our qualitative analysis we identified five categories within this theme: experience and awareness of researchers; interests and rights of research subjects; social value of research; organization of review; and problems in the review process. For each category we identified a set of subcategories as summarized in Additional file 3: Table S3 and referenced to the specific guidelines.

*Experience and awareness of researchers* was mentioned in many research ethics guidelines for disaster settings, and our analysis showed five subcategories for this category: cultural sensitivity of researchers (in five guidelines); awareness of impact of research (in three guidelines); conflicts of interest (in four guidelines); training in research ethics (in four guidelines); and professional competence of researchers (in three guidelines).

Cultural sensitivity of researchers includes the way authors discussed that research agendas and interventions proposed by researchers in disaster settings often are based on a Western perspective which may impact negatively on local populations [14, 15]. Additionally, specific methods and instruments may have limited validity when used in oral cultures [14]. As a result, guidelines pointed out that research protocols should discuss how cultural factors have “informed the research design and its implementation, and how these factors will be evaluated and by whom during the project” [19]. Researchers also should be aware of implicit messages given as a result of selection of specific research areas [10]. This issue included statements about the necessity for researchers to be aware of the possible indirect and direct impact of their research [10, 13], and the “ability to anticipate adverse reactions and facilitate appropriate interventions” [15].

Within the ethics review process, evaluation of conflicts of interest was included, but no specific aspects were linked to disaster settings. Training in research ethics was mentioned as an important aspect by many guidelines, including that RECs be required to ensure that researchers complete an ethics module on doing research in disaster situations. Some guidelines also mentioned that research support staff should be provided ethics training [14, 19].

*Interests and rights of research subjects* was one of the central and most frequently addressed aspects of the guidelines included in our review. This category included seven subcategories: balancing the need for scientific evidence with possible harm from the research (in ten guidelines); minimal risk requirement (in four guidelines); justice in selection of participants (in eight guidelines); potential for overburdening research subjects (in six guidelines); provisions for confidentiality and privacy protection (in nine guidelines); regulation of transfer of biological material (in five guidelines); and application of standard of care (in three guidelines).

Almost all guidelines emphasized that research in disaster settings must carefully balance the need for scientific evidence and the need to protect research subjects from possible harm from the research itself. Some guidelines emphasized the strong ethical mandate to do research in disaster settings “to prevent further death and illness in present or future disasters” [21], with some suggesting it might be unethical not to do such research [11]. At the same time, guidelines do not provide specific methods for evaluating risks and benefits. One possible approach was applying the minimal risk requirement suggested in three guidelines: for research in refugee populations [16], with biomedical research [11], and in clinical research [15]. One guideline proposed the minimal risk requirement as a precondition for expedited review [20].

Justice in selection of participants means “making political and ethical choices about which voices are heard and whose knowledge counts” [10]. The main requirement was that research participants should be chosen based on scientific reasons and not on any other reason, like accessibility, cost, gender or malleability [15]. Allden et al. mentioned examples where local governments suggest not interviewing marginalized groups or certain religious communities [14]. Jennings and Arras pointed out two other concerns regarding justice in selection of research subjects: where the participants and their community should benefit from the results of a successfully conducted trial (a negative example would be a trial of an expensive drug conducted in a poor country where the drug would never be affordable) and where the participants are from a vulnerable population that might make them more easily abused or exploited [21].

Another subcategory within the interests and rights of research subjects was the potential for overburdening research subjects with multiple or repetitive studies or by adding research burdens to their traumatic experiences. As a possible solution for this problem, some guidelines pointed to the importance of dissemination of research results among researchers to share information and avoid duplication of effort [14, 15].

As for REC review with other types of research, guidelines emphasized that disaster research protocols should develop and apply explicit provisions for confidentiality and privacy protection. In disaster research, this applies not only at the level of individual participants, but also at the community level. Another specific issue in disaster settings was extensive media attention that can lead to breaches of confidentiality [13, 14].

Regulation of transfer of biological material was addressed in five guidelines. The Médecins Sans Frontières (MSF) guideline included the most detailed discussion of this topic and stated that it should be based on a commitment “to serve the beneficiaries of a humanitarian medical intervention, not the interests of third parties such as the developers of commercial tests” [20]. Transfer of biological material should not only follow existing legal requirements, but also apply ethically acceptable consent procedures, clearly explaining the purposes of collecting and storing samples, ownership of data, intellectual property, and other issues [15].

Another subcategory included in the guidelines was the application of standard of care in disaster settings and the justification of possible alterations [8, 9, 21]. The general standard of care debate in research ethics addresses questions about the kind of medical care researchers owe to research participants for treating their condition or disease under study or other medical needs arising during participation in research. However, the guidelines found in this review mentioned this topic very generally and did not discuss it in ways specific to disaster settings.

*Social value of research* was another category that frequently arose and contained six subcategories to be addressed in REC review: potential application to future disaster situations (in five guidelines); research that cannot be pursued in a non-disaster context (in six guidelines); direct or indirect benefit to individuals or community (in eleven guidelines); not draining resources for relief (in two guidelines); involvement of local researchers and/or community (in nine guidelines); and post-research obligations (in four guidelines).

The first subcategory was the potential application of the research results to future disaster situations. Guidelines stated that it would be ethically questionable to perform research during disasters that target crisis events of extremely low probability [21]. To ensure that results will have future application, researchers need “to systematically map existing and relevant evidence pertaining to disasters” [15].

Six guidelines stated that ethically acceptable research in disaster settings should be research that cannot be pursued in a non-disaster context. Curry et al. mentioned as an example that “the implementation of a clinical drug or vaccine trial in a refugee camp for reasons of convenience – subjects easy to find, no loss-to-follow-up, etc. – is clearly unacceptable because the same research could be done in situations where participants have much greater agency” [19].

Direct or indirect benefit to individuals and/or communities was another criterion included in evaluating research proposals for disaster settings. Some guidelines referred to “important direct benefit” [16], while others stated that the benefit might be indirect if an agreement is reached between the community and the researcher [11]. Likewise, the MSF guidelines stated that sometimes it is acceptable to test “an intervention that is too expensive at the outset of the research to be made immediately available to everyone who needs it […] if there are good reasons to expect a considerable price drop and if MSF initiates advocacy and lobbying efforts at the same time” [20].

Two guidelines included a provision that RECs should also evaluate whether the proposed research will drain funds, resources or necessary personnel devoted to immediate disaster relief. If research risks draining resources for relief “such research should either not be conducted in the present circumstance or additional funds or personnel should be devoted to the research in a way that would not threaten or undermine the primary goals of crisis response” [21]. Sumathipala et al. added that the main reason for this problem is that research activities are often “uncoordinated and poorly integrated with humanitarian relief operations” [15].

According to many guidelines, the involvement of local researchers and/or communities was a very important criterion for ethical disaster research. Representatives of the community (ideally, representatives of research participants) should take part in the planning and implementation of research projects. Some guidelines mentioned particular forms of participation, e.g., community-based participatory research [14, 21] or involving representatives of local unaffected communities [17]. At the same time, other guidelines indicated that involvement of the community “may seem impossible in the chaos and confusion post-disaster” [17]. Equally important is the involvement of local researchers as equal research partners, as well as involvement of local experts and lay persons as members of RECs and advisory boards.

Post-research obligations included feedback of research results to research participants [10, 13] and the general public [19], as well as “sharing downstream benefits from the research”, e.g., “invention of new medical procedures or intervention strategies” or “intellectual property (IP), to new or improved commercial products or processes” [19].

*Organization of ethics review* in disaster settings is challenging and complicated. The included guidelines offered several possible ways to address this problem that have been organized into the following subcategories: centralization of review (in seven guidelines); conditions for full and expedited review (in seven guidelines); alternative review mechanisms (in four guidelines); “just-in-case protocols” (in four guidelines); and proportionality of review (in one guideline).

Centralization of ethics review by establishing a new disaster-focused REC (national, regional, etc.) or by delegating reviews of disaster research to an existing REC was suggested as having certain advantages, e.g., “maximize the knowledge obtained from the research, coordinate the numerous studies, minimize the burden on research subjects, and attend to simultaneous needs for acquiring new knowledge and clinical treatment” [13]. Another form of centralized review was MSF’s ethics review board set up in 1999 specifically for MSF research [9]. However, empirical analysis of the effects of centralization of review has not been conducted.

Clear rules for full and expedited review might be helpful for the organization of such reviews. In most guidelines, expedited review was deemed sufficient if the research study carried minimal risks to participants and did not include any worrisome or novel ethical issues. Guidelines also mentioned alternative review mechanisms not limited to traditional standard review procedures, e.g., development of preparedness plans for researchers, institutions and RECs to “proactively address basic operational questions” [18], “rolling” or contemporaneous review for protocols or parts of protocols [8], individual review by the chair of the board or the chair’s delegate, and prioritization of protocols by a specific triage committee [12]. At the same time, guidelines included a warning that “any exemptions to normal practices in research ethics review should be rare and should require a high level of justification” [12]. Another form of alternative review suggested in guidelines was “just-in-case protocols” which includes planning and reviewing at least a general outline of a research study or a “generic” protocol in advance of a disaster. This proposal also included creating a pre-disaster repository of these protocols or protocol parts. One guideline also mentioned proportionate review, defined as a form of review “intended to reserve the most intense scrutiny, and correspondingly more protection, for the most ethically challenging research” [12].

*Problems in the review process* included three subcategories: risk of bureaucracy in the review process (in three guidelines); lack of guidelines for research in disaster settings (in two guidelines); and distinction between research and non-research (in one guideline). Overly burdensome bureaucracy and undue delays in the review process were mentioned in three guidelines and appeared as a problem often faced by researchers. Another problem was a lack of specific guidelines for research in disaster settings in existing general research ethics guidelines like the Helsinki Declaration and CIOMS guidelines.^1^ The third problem raised was the lack of a clear distinction between research and non-research, particularly as it relates to the boundary between public health-oriented research and practice [8].

## Discussion

To increase the quality of disaster response activities and interventions, additional research in disaster settings is needed. Many research papers and other documents note that such research often raises specific ethical challenges that should be addressed and adequate guidance should be developed. Our paper is a descriptive study that reviews the existing ethical guidelines on the ethics of research in disasters. Our aim was to identify, describe and compare disaster research ethics guidelines. A thorough critical analysis of these guidelines is warranted, but would require its own article. However, a limited critical analysis will be presented here, along with some proposals for the development of further ethical guidance for disaster research. Our systematic search identified 14 guidelines (in English) that met our inclusion criteria and are applicable to diverse research activities in different settings. Disaster research covers a wide variety of research types and has several ethically relevant characteristics in common with research in public health emergencies, research in conflict zones, clinical research in emergency settings, research in low- and middle-income countries, or research conducted in resource poor settings.

The scope of the guidelines we found was rather narrow. Most of the analyzed documents did not attempt to give researchers and other stakeholders a comprehensive overview of how to proceed ethically in all types of research and in all types of disasters, but rather focused on particular research activities in specific settings and with distinct populations, such as conflict zones, refugee populations, and humanitarian settings. A tension exists here because disaster research is unavoidably context and time sensitive, making generalized guidance less applicable. While taking this into account, a need remains to develop a more comprehensive set of guidelines based on the ethical issues identified here as commonly relevant to many forms of disaster research. Other issues may need to be included also.

One of the two core themes that emerged was vulnerability of research subjects. CIOMS guidelines refer to vulnerability as “a substantial incapacity to protect one’s own interests”, and accordingly state that “special provision must be made for the protection of the rights and welfare of vulnerable persons” [22]. In a paper by O’Mathúna, it is noted that vulnerability in a disaster research setting presents additional duties for researchers [5]. Chung et al. proposed that “the individuals and communities affected by declarations of a state of emergency or disaster should be considered ‘vulnerable subjects’ for the purposes of human subjects research”, which would enable the use of current research guidelines in disaster settings [17]. This approach to vulnerability is not based on a lack of decision making capacity, but rather on the effects of the disaster situation on an individual (participant) or a group (of participants).

One of the guidelines included in this review questioned the applicability of the broad approach to vulnerability to disaster settings because it might stereotype and disempower research subjects due to the specificity and complexity of a disaster situation [13]. Although the concept of vulnerability is raised in research to ensure special protection for vulnerable participants, further clarification of the concept would help to guide RECs and ensure appropriate protections are put in place [23, 24]. Empirical assessment is needed to determine if this approach is sufficient to ensure such protection in a disaster setting.

Beyond vulnerability as a core theme in disaster settings, the analyzed guidelines discussed and attempted to raise awareness about specific risks that disaster research might pose for participants. According to the guidelines, issues that require careful consideration in the design of research protocols and during REC review are the higher risk of therapeutic misconception, the potential for exploitation, manipulation, or re-traumatization of research participants, and also the issue of compromising care or relief for research. In some specific disaster situations, for example in armed conflict zones, even the simple presence of foreign researchers could pose an additional risk to the local community. The need to take such risks into account, especially unintended ones, was one of the most frequently raised ethical issues. The actual risks will vary by research study, but further guidance is need on how to identify disaster-specific risks, especially unintended ones. This highlights the importance of involving experienced researchers and local representatives (of participants and their communities, as well as local researchers) in the design, review and implementation of disaster research. Applying specific study designs that ensure community participation in disaster research (e.g., community based participatory research) should be considered, where appropriate, to ensure effective collaboration.

Most guidelines discussed the need for specific procedures for adequate informed consent. Generally, informed consent was seen as a necessary but also challenging requirement in situations where language and cultural barriers could be determining factors, as well as where the decision-making capacities of participants could have been impacted by disasters. A repository of innovative and evidence-based approaches to informed consent would be very valuable.

Nearly all guidelines described the independent and prior assessment of disaster research by an ethics committee as an important ethical requirement. However, we found great heterogeneity in the specific recommendations for organization of the review, for the assessment process, and also for the specificities of the risk/benefit assessments. Some guidelines viewed protocols as ethically acceptable only if they had a direct benefit for participants, and if the research could not be performed in non-disaster settings. In contrast, other guidelines would not prohibit such research if it posed minimal harm and had a considerable benefit to society. Most guidelines considered the usual REC review procedures as unsuitable in disaster settings. Thus, various innovations were suggested regarding the organization of independent ethics review for disaster research. Empirical research is needed to determine the effectiveness of these approaches in improving the ethical dimensions of disaster research so that its ethical review can become more evidence-based. International and national stakeholders responsible for research ethics approval and review should evaluate the appropriateness of their current ethics approval procedures and their suitability for disaster research.

Although mentioned by only one guideline, the proper coordination of research activities with humanitarian relief operations is another important point to consider. In a disaster setting, a proper coordination center might provide a way to involve local researchers and the local community. Such a center could reduce significantly the duplication of research activities, and adequately assess the potential conflict between research and treatment or aid. It could also give proper consideration to ethical perspectives on benefit sharing with the local community and research participants. Many guidelines focus on the ethical issues commonly addressed in research ethics, but the ethical issues distinct to research in resource poor countries or public health practice need to be incorporated more fully.

Some of the guidelines, like those developed by the Working Group on Disaster Research and Ethics, were explicitly based on a retrospective assessment of a concrete disaster research experience [15], while others remained obscure regarding the evidence base and motivations for their development. This highlights the need for more empirical research and evidence regarding the ethics of research in disaster settings. Such an example is the Post-Research Ethics Analysis [25] project that attempts to collect and assess concrete, real world research experiences and ethical challenges faced by researchers and other stakeholders that could further support the development and evaluation of such guidance documents.

## Conclusions

Our study found 14 guidelines that might be applied in disaster research settings. Most guidelines referred to vulnerability of research subjects as a central issue, but defined the concept in different ways. The role of RECs was widely acknowledged as challenging in such circumstances. It seems especially important for RECs to consider the potential need for non-standard ethics review procedures for disaster research settings. It is also essential to ensure appropriate dissemination of disaster research results among researchers to share information, and avoid duplication of effort and overburdening of research subjects.

We also found some gaps in the studied guidelines, where further work is certainly needed. Emerging guidelines should include practical suggestions regarding how to weigh conflicting principles, or references to such sources, which could support researchers in their work. Given the tension noted above between generalized and specific approaches to guidance, such practical decision-making tools will be essential. The evidence base of the studied guidelines is rather weak and diverse. Most guidelines were based on some personal experiences, unique situations, or NGO practices. Empirical evidence is urgently needed to support the statements and requirements included in research ethics guidelines. These include the prevalence of ethically significant scenarios, and a typology of ethical issues, including, for example, vulnerability, re-traumatization, or lack of local REC approval. Disaster researchers and the RECs who review their protocols should include projects to evaluate how well the ethical issues are addressed in the research and by following REC recommendations. Particular attention should be given to assessing participants’ perceptions of how ethics is addressed in specific projects.

National RECs and international networks of RECs, or their professional associations, should reconsider their standard recommendations and procedures in light of the challenges posed by disaster situations. A comprehensive guideline for disaster research ethics that takes account of the different types of research methods, contexts and populations would be very helpful. Models for proper coordination centres in disaster settings that could be responsive to the identified research ethics challenges could be advantageous. Beyond the clear need for further work on disaster research ethics guidelines, we also see that it is important that specific educational resources for disaster research ethics training be developed and disseminated.

## Additional files

Additional file 1: Table S1. List of Disaster Research Ethics Guidelines. A list of the 14 research ethics guidelines included in this review providing their year of issue, issuing organization, reference, reach and scope. (DOCX 27 kb)
Additional file 2: Table S2. The categories and subcategories within the core theme “Vulnerability”. The analysis identified two core themes, one being Vulnerability. This table lists the four categories identified within this theme, and the subcategories within each of these categories. (DOCX 23 kb)
Additional file 3: Table S3. The categories and subcategories within the core theme “research ethics committee (REC) review process”. The analysis identified two core themes, one being Research Ethics Committee Review. This table lists the five categories identified within this theme, and the subcategories within each of these categories. (DOCX 24 kb)

## Abbreviations

CCM: Constant comparative method
CIOMS: Council for International Organizations of Medical Sciences
IRB: Institutional review board
MSF: Médecins sans frontières
REC: Research ethics committee
WHO: World Health Organization

## Footnotes

1 This will be corrected in the forth-coming revision of the CIOMS guidelines where Guideline 20 specifically addresses disaster research (http://www.cioms.ch/).
