# Supplementary material for: Research in disaster settings: a systematic qualitative review of ethical guidelines
Source: BMC Med Ethics. 2016 Oct 21;17:62. doi: 10.1186/s12910-016-0148-7 (PMC5073437; doi:10.1186/s12910-016-0148-7)
Supplement: Additional file 1: Table S1. — List of Disaster Research Ethics Guidelines. A list of the 14 research ethics guidelines included in this review providing their year of issue, issuing organization, reference, reach and scope. (DOCX 27 kb) [file 12910_2016_148_MOESM1_ESM.docx]

Supplementary Table 1. List of Disaster Research Ethics Guidelines

| **No.** | **Year** | **Issuing organization/ institution/event** | **Title and reference** | **Reach** | **Scope** |
| --- | --- | --- | --- | --- | --- |
| 1. | 2000 | International NGO Training and Research Centre | Goodhand J. Research in conflict zones: ethics and accountability. Forced Migr Rev. 2000;8:12–5. | international | social science research in conflict zones |
| 2. | 2001 | Individual researcher | Leaning J. Ethics of research in refugee populations. Lancet. 2001;357(9266):1432-3. | international | different types of research in refugee populations and internally displaced peoples |
| 3. | 2004 | A meeting organized by the New York Academy of Medicine and the National Institute of Mental Health | Collogan LK, Tuma F, Dolan-Sewell R, Borja S, Fleischman AR. Ethical issues pertaining to research in the aftermath of disaster. J Trauma Stress. 2004;17(5):363–72. | national (US) | different types of research on trauma-exposed participants in the aftermath of disaster |
| 4. | 2006 | Indian Council of Medical Research | Indian Council of Medical Research. ICMR’s ethical guidelines for biomedical research on human participants. 2006. | national (India) | general guidelines on ethics in medical research including a chapter on research in disaster settings |
| 5. | 2008 | US Centers for Disease Control and Prevention,  Advisory Committee to the Director | Jennings B, Arras J. Ethical guidance for public health emergency preparedness and response: highlighting ethics and values in a vital public health service. Centers for Disease Control and Prevention. 2008. | institutional (US Centers for Disease Control and Prevention) | general ethical guidance for public health emergency response during or immediately after disaster events including research ethics guidelines |
| 6. | 2008 | Written by group of researchers | Chung B, Jones L, Campbell LX, Glover H, Gelberg L, Chen DT. National recommendations for enhancing the conduct of ethical health research with human participants in post-disaster situations. Ethn Dis. 2008;18(3):378–83. | national (US) | health-related human subject research in post-disaster situations |
| 7. | 2009 | Médecins Sans Frontières | Schopper D, Upshur R, Matthys F, Singh JA, Bandewar SS, Aasim A, van Dongen E. Research ethics review in humanitarian contexts: the experience of the independent ethics review board of Médecins Sans Frontières. PLoS Med. 2009;6(7):e1000115. | international | ethics review of health research in humanitarian contexts |
| 8. | 2009 | Harvard Humanitarian Action  Summit, The Working Group on Mental Health and Psychosocial  Support | Allden K, Jones L, Weissbecker I, Wessells M, Bolton P, Betancourt T, Hijazi Z, Galappatti A, Yamout R, Patel P, Sumathipala A. Mental health and psychosocial support in crisis and conflict: report of the Mental Health Working Group. Prehospital Disaster Med. 2009;24 Suppl 2:s217–27. | international | mental health and psychosocial  research in humanitarian settings |
| 9 | 2010 | World Health Organization | World Health Organization. Research ethics in international epidemic response: WHO technical consultation, Geneva, Switzerland, 10-11 June 2009: meeting report. 2010. | international | research during international epidemic response and public health emergencies |
| 10. | 2010 | Canadian Medical Association | Tansey CM, Herridge MS, Heslegrave RJ, Lavery JV. A framework for research ethics review during public emergencies. Can Med Assoc J. 2010;182(14):1533-7. | national (Canada) | research ethics review during public emergencies |
| 11. | 2010 | Working Group on Disaster Research and Ethics | Sumathipala A, Jafarey A, De Castro L, Ahmad A, Marcer D. Ethical issues in post-disaster clinical interventions and research: a developing world perspective. Key findings from a drafting and consensus generation meeting of the Working Group on Disaster Research and Ethics (WGDRE) 2007. Asian Bioeth Rev. 2010;2:124–42. | international | post-disaster clinical interventions and research |
| 12. | 2013 | Médecins Sans Frontières | MSF Ethics Review Board standard operating procedures. 2013. | organizational (MSF) | ethics review in natural and man-made disaster situations |
| 13. | 2014 | Canadian Institutes of Health Research, Natural Sciences and Engineering Research Council of Canada, Social Sciences and Humanities Research Council of Canada. | Tri-Council policy statement: ethical conduct for research involving humans. Second edition. 2014. | national (Canada) | ethics review during publicly declared emergencies |
| 14. | 2014 | Enhancing Learning and Research for Humanitarian Assistance (ELRHA) | Curry DR, Waldman RJ, Caplan AL. An Ethical Framework for the development and review of health research proposals involving humanitarian contexts. 2014. | international | health research involving humanitarian contexts |
