# Supplementary material for: Research in disaster settings: a systematic qualitative review of ethical guidelines
Source: BMC Med Ethics. 2016 Oct 21;17:62. doi: 10.1186/s12910-016-0148-7 (PMC5073437; doi:10.1186/s12910-016-0148-7)
Supplement: Additional file 2: Table S2. — The categories and subcategories within the core theme “Vulnerability”. The analysis identified two core themes, one being Vulnerability. This table lists the four categories identified within this theme, and the subcategories within each of these categories. (DOCX 23 kb) [file 12910_2016_148_MOESM2_ESM.docx]

Supplementary Table 2. The categories and subcategories within the core theme “Vulnerability”

| **Categories** | **Subcategories** | **Guideline number (as per Table 1)** |
| --- | --- | --- |
| **Vulnerability as a concept** | | |
|  | definitions of vulnerability | 3;5;6;9 |
|  | reasons for vulnerability | 2;3;6;11;13;14 |
|  | gaps in the existing guidelines | 2;3 |
| **Risks and burdens** | | |
|  | physical harm | 1;2;3;8;10;13;14 |
|  | re-traumatization | 1;3;6;7;11 |
|  | manipulation | 2;8 |
|  | exploitation | 1;2;5;7;8;9;11;14 |
|  | unrealistic expectations | 1;3;5;6;7;8;9;11 |
|  | stigmatization | 8;11 |
| **Risk management** | | |
|  | accountability and monitoring of research | 3;6;7;8;9;10;11;12;14 |
|  | avoiding over- or underestimation of risks | 3;5;6 |
|  | need for empirical evidence on risk | 3;5;6 |
|  | providing psychological support to research subjects | 3;5;6;8 |
|  | quality of informed consent | 1;3;7;8;11;14 |
|  | evaluation of power relationships between researchers and subjects | 1;8 |
| **Decisional capacity of research subjects** | | |
|  | factors diminishing decisional capacity | 3;5;13;14 |
|  | underestimation of decisional capacity | 3;11 |
|  | need for a specific procedure for informed consent | 3;5;6;7;13 |
