# Supplementary material for: Research in disaster settings: a systematic qualitative review of ethical guidelines
Source: BMC Med Ethics. 2016 Oct 21;17:62. doi: 10.1186/s12910-016-0148-7 (PMC5073437; doi:10.1186/s12910-016-0148-7)
Supplement: Additional file 3: Table S3. — The categories and subcategories within the core theme “research ethics committee (REC) review process”. The analysis identified two core themes, one being Research Ethics Committee Review. This table lists the five categories identified within this theme, and the subcategories within each of these categories. (DOCX 24 kb) [file 12910_2016_148_MOESM3_ESM.docx]

Supplementary Table 3. The categories and subcategories within the core theme “REC review”

| **Categories** | **Subcategories** | **Guideline number (as per Table 1)** |
| --- | --- | --- |
| **Experience and awareness of researchers** | | |
|  | cultural sensitivity of researchers | 1;4;8;11;14 |
|  | awareness of impact of research | 1;3;11 |
|  | conflicts of interest | 4;11;12;14 |
|  | training in research ethics | 6;8;9;14 |
|  | professional competence of researchers | 11;12;14 |
| **Interests and rights of research subjects** | |  |
|  | balancing the need for scientific evidence with possible harm from the research | 2;3;4;5;7;8;10;11;12;14 |
|  | minimal risk requirement | 2;4;7;11 |
|  | justice in selection of participants | 1;2;5;6;8;11;12;13 |
|  | potential for overburdening research subjects | 3;5;6;7;8;11 |
|  | provisions for confidentiality and privacy protection | 1;3;4;6;8;9;11;12;14 |
|  | regulation of transfer of biological material | 4;7;9;11;12 |
|  | application of standard of care | 5;9;12 |
| **Social value of research** | |  |
|  | potential application to future disaster situations | 4;5;6;11;14 |
|  | research that cannot be pursued in a non-disaster context | 5;6;7;8;13;14 |
|  | direct or indirect benefit to individuals or community | 1;2;3;4;5;6;7;8;11;12;14 |
|  | not draining resources for relief | 5;11 |
|  | involvement of local researchers and/or community | 3;4;5;6;8;9;11;12;14 |
|  | post-research obligations | 1;3;12;14 |
| **Organization of ethics review** | | |
|  | centralization of review | 3;5;6;7;8;11;12 |
|  | conditions for full and expedited review | 4;5;7;9;10;11;12 |
|  | alternative review mechanisms | 5;9;10;13 |
|  | "just-in-case protocols" | 5;7;9;12 |
|  | proportionality of review | 10 |
| **Problems in the review process** | | |
|  | risk of bureaucracy in the review process | 3;5;9 |
|  | lack of guidelines for research in disaster settings | 6;11 |
|  | distinction between research and non-research | 9 |
